# A Nomogram Model to Predict Prognosis of Patients With Genitourinary Sarcoma

**DOI:** 10.3389/fonc.2021.656325

**Published:** 2021-04-16

**Authors:** Linde Li, Jiayu Liang, Turun Song, Saifu Yin, Jun Zeng, Qiang Zhong, Xiaobing Feng, Zihao Jia, Yu Fan, Xianding Wang, Tao Lin

**Affiliations:** ^1^ Department of Urology, Institute of Urology, West China Hospital, Sichuan University, Chengdu, China; ^2^ Organ Transplantation Center, West China Hospital, Sichuan University, Chengdu, China

**Keywords:** genitourinary sarcoma, histology, surgery, chemotherapy, prognosis, nomogram

## Abstract

**Objectives:**

The aim of this study is to evaluate the significant factors influencing the overall survival (OS) and recurrence free survival (RFS) and make an attempt to develop a nomogram for predicting the prognosis of patients with genitourinary sarcoma (GS).

**Methods:**

Data on adult GS from 1985 to 2010 were collected. The impact of clinical factors on OS and RFS were estimated by Kaplan–Meier (KM) analysis, and differences between groups were analyzed by the log-rank test. To establish a nomogram, all patients were randomly divided into a training set (n = 125) and a testing set (n = 63). Cox proportion hazard model was utilized to assess the prognostic effect of variables. Then, a nomogram was established to estimate 1-, 3-, and 5-year OS based on Cox regression model. Subsequently, the nomogram was validated by a training set and a validation set.

**Results:**

A total of 188 patients were enrolled into our study. Male patients with bladder sarcoma had better OS rather than RFS when stratified by gender (P = 0.022). According to histological subtypes, patients with leiomyosarcoma (LMS) undergoing chemotherapy were associated with favorable OS (P = 0.024) and RFS (P = 0.001). Furthermore, LMS in kidney sarcoma were associated with lower recurrence rate in comparison to rhabdomyosarcoma (RMS) (P = 0.043). Margin status after surgical excision markedly influenced the OS and RFS of GS patients and negative margins presented optimal prognosis. Chemotherapy was associated with improved OS for patients without surgery (P = 0.029) and patients with positive margins (P = 0.026). Based on the multivariate analysis of the training cohort, age, gender, surgery status, histological subtype, and chemotherapy were included in our nomogram for prediction of OS. The nomogram had sufficient power with concordance index (C-index) of OS: 0.770, 95%CI: 0.760–0.772 and area under curve (AUC) of OS: 0.759, 95%CI: 0.658–0.859 in the training set and with C-index of OS: 0.741, 95%CI: 0.740–0.765, and AUC of OS: 0.744, 95%CI: 0.576–0.913 in the validation set.

**Conclusions:**

Adults GS is a group of extremely rare tumors with poor prognosis. Of all histological types, LMS is sensitive to chemotherapy. We highlighted the cardinal role of surgical resection and the importance of achieving negative margins. We identified the efficacy of chemotherapy for patients with positive margins and those without surgery as well. A nomogram is validated as an effective tool predicting short-term outcomes.

## Introduction

Soft tissue sarcomas (STSs) are a group of rare and heterogeneous neoplasm that originate from the embryo mesoderm and make up 1–2% of all adult malignancies (1). There are more than 100 different histological and molecular subtypes of STS, each with unique clinical, prognostic features. In 2017, there were approximately 10,000 patients diagnosed with STS in the USA, accounting for approximate 0.73% of new cancer cases (2). Genitourinary sarcomas (GSs) occur rarely in adults, with less than 5% of STS (3). Thus, clinical characteristic, treatment, and survival of patients with GS are predominantly based on the knowledge from studies on pediatric or other sites (4).

The studies on the prognosis of GS in the literature are limited. A study reported 131 cases from July 1977 to July 2003 suggested that the clinically relevant prognostic factors and clinical characteristics included local disease at presentation, complete tumor resection, tumor grade, size, local, and histological subtype (1). Another study using SEER 18 database found tumor location and histological types significantly influenced overall survival (4). We previously reported that age, tumor grade, margin status, and chemotherapy were independently associated with survival of GS patients (5). It was common that complete resection played a crucial role in the treatment of GS. However, whether GS patients could benefit from chemotherapy combined with radiotherapy or alone remains unclear. Therefore, it is necessary to characterize the role of chemotherapy and radiotherapy in the management of GS patients.

As a reliable and convenient prognostic tool, the nomogram has been widely adopted to predict the outcomes of an individual and benefit for patients and clinicians (6). A nomogram can predict the prognosis in certain patients based on molecular features and crucial prognostic factors and explain the numerical probability of clinical outcomes (7). There are three nomograms predicting sarcoma-specific death, local regional recurrence, and OS for patients with extremity STS and four nomograms predicting OS for patients with retroperitoneal sarcoma, which were proven to be reliable and effective (8–14). However, there is lack of nomogram predicting survival for GS patients.

Clinical information of GS patients treated at the West China Hospital, Sichuan University, Chengdu, Sichuan, China from 1985 to 2010 was extracted. Based on the clinicopathological information of 188 patients, this study assessed the impact of gender, histological subtype, surgery, and adjuvant therapy on OS and RFS, developed a nomogram model to predict OS of GS patients and determined the accuracy of the nomogram model and independently clinicopathological features which were related to OS of GS patients.

## Methods

### Patient Selection

Data on consecutive adult patients (over a period of 16 years) who had histologically proven primary genitourinary sarcoma (GS) was collected in the medical archives of our institution from June 1985 to June 2010.

The inclusion criteria included: (1) histologically proven primary GS; (2) diagnosed from June 1985 to June 2010; (3) primary site located in genitourinary tract, such as the paratesticular region, kidney, prostate, bladder, penis, seminal vesicle, ureter, and urethra; (4) complete follow-up. The excluded criteria were as follows: (1) survival months < one month; (2) multiple primary cancer; (3) the female genital tract, and genitourinary organs invaded by retroperitoneal and pelvic sarcomas.

### Prognostic Variables

Data were extracted from the West China Hospital, Sichuan University, Chengdu, Sichuan, China on patient age, gender, primary organ, symptom at presentation, symptom duration, tumor size, histological subtype, metastasis at entry, grade, surgery status, chemotherapy, radiotherapy, recurrence time, and survival time. The sarcoma was considered as unclassified when histological subtype could not be identified. Tumor grade was divided into low grade (Grade 1) and high grade (Grades 2 and 3) according to the French Federation of Cancer Center System Grading Scheme for Adults Sarcoma, as suggested by Deyrup et al. (15). Surgical margins were considered as negative by a consensus of both operation and pathological records if microscopically and macroscopically residual tumor was absent. Follow-up data were collected in outpatient department or by telephone interviews.

The influence of gender, histological subtype, surgery status, chemotherapy and radiotherapy on OS and RFS were estimated by Kaplan–Meier (KM) analysis, and differences between groups were analyzed by the log-rank test. Univariate and multivariate Cox regression analysis were subsequently utilized to identify significant factors affecting OS and RFS of GS patients.

### Nomogram Construction and Validation

All patients were randomly divided into training set (n = 125) and validation set (n = 63) at a ratio of 2:1 through the “caTools” package in R software. These characteristics between two sets were compared by the chi-square test. Fishing exact test or adjusted chi-square test were also used if needed. Univariate analysis and multivariate stepwise Cox regression analysis were performed to identify risk factors affecting OS in the training set. Using these identified risk factors, a nomogram was developed to predict 1-, 3- and 5-year OS in GS patients.

According to the risk scoring model based on the nomogram, the risk score was calculated for each patient in the training and validation sets. All patients were classified into a high risk group and a low risk group with regard to median risk score (high risk: median risk score >0; low risk: median risk score ≤0). The OS of patients between two groups was compared by KM survival curve.

The nomogram was validated in the training set and validation set. We evaluated the predictive performance of the nomogram by C-indices and AUC in receiver operating characteristic (ROC) curve. The consistency of actual survival with predicted outcomes was compared by calibration curves in the training and validation sets. All statistical analyses were performed by the R software version 4.0 (http://www.r-project,org/). P-value of <0.05 was expected as statistically significant.

## Results

### Patient Characteristic

188 patients were identified in the study period. The clinical characteristics of the study population were summarized in **Table 1**. GS sarcoma was more likely to locate in paratestis (29.8%); gender disparity was obvious (male 75.5%); more than half of patients aged less than 50 years; diameter of tumor was beyond 5 cm in 79.8% of all patients; the most common histological subtype was LMS; the majority of patients had no metastasis at presentation; patients with high-grade tumor accounted for 80.9% of study population; more than half of patients received surgery; 79.1% of patients with surgery had negative margins and 33 patients did not undergo surgery; of all, 71.7% of patients received chemotherapy and 36.7% of patients underwent radiotherapy. At the end of follow-up, RFS rate was 35.5%, and OS rate was 19.8%. Chemotherapy and radiotherapy regimens were previously reported (5).

**Table 1:** Clinical characteristics of patients with genitourinary sarcoma in the present study.

Characteristics	Value (%)
**PRIMARY ORGAN**	
Bladder	40 (21.3)
Kidney	49 (26.1)
Other	13 (6.9)
Paratesticular	56 (29.8)
Prostate	30 (16.0)
**GENDER**	
Female	46 (24.5)
Male	142 (75.5)
**AGE**	
>50 years	75 (39.9)
≤50 years	113 (60.1)
**PRESENTING SYMPTOMS**	
Asymptomatic	9 (4.8)
Symptomatic	179 (95.2)
**SYMPTOMATIC DURATION**	
>1month	116 (61.7)
≤1month	72 (38.3)
**TUMOR SIZE**	
>5 cm	150 (79.8)
≤5 cm	38 (20.2)
**HISTOLOGICAL SUBTYPE**	
Liposarcoma	38 (20.2)
Leiomyosarcoma	77 (41.0)
Other	37 (19.7)
Rhabdomyosarcoma	36 (19.1)
**METASTASIS_AT_ENTRY**	
no	140 (74.5)
yes	48 (25.5)
**GRADE**	
high	152 (80.9)
low	36 (19.1)
**SURGERY_STATUS**	
Surgery and margin negative	106 (63.5)
No Surgery	33 (19.8)
Surgery and margin positive	28 (16.8)
**CHEMOTHERAPY**	
no	51 (28.3)
yes	129 (71.7)
**RADIOTHERAPY**	
no	114 (63.3)
yes	66 (36.7)
**RECURRENCE**	
no yes **SURVIVAL STATUS** Alive Dead	41 (35.3) 75 (64.7) 34 (19.8) 138 (80.2)

Other in Primary organ included four genitourinary sites: ureter, seminal vesicles, and urethra. Other in histological subtype included Ewing’s sarcoma, primitive neuroectodermal tumor, synovial sarcoma, angiosarcoma, fibrosarcoma, malignant peripheral sheath tumor, myo-fibroblastic tumor, osteosarcoma, chondrosarcoma, and undifferentiated sarcoma.

### The Effect of Gender on OS and RFS of GS Patients

**Figure 1:**
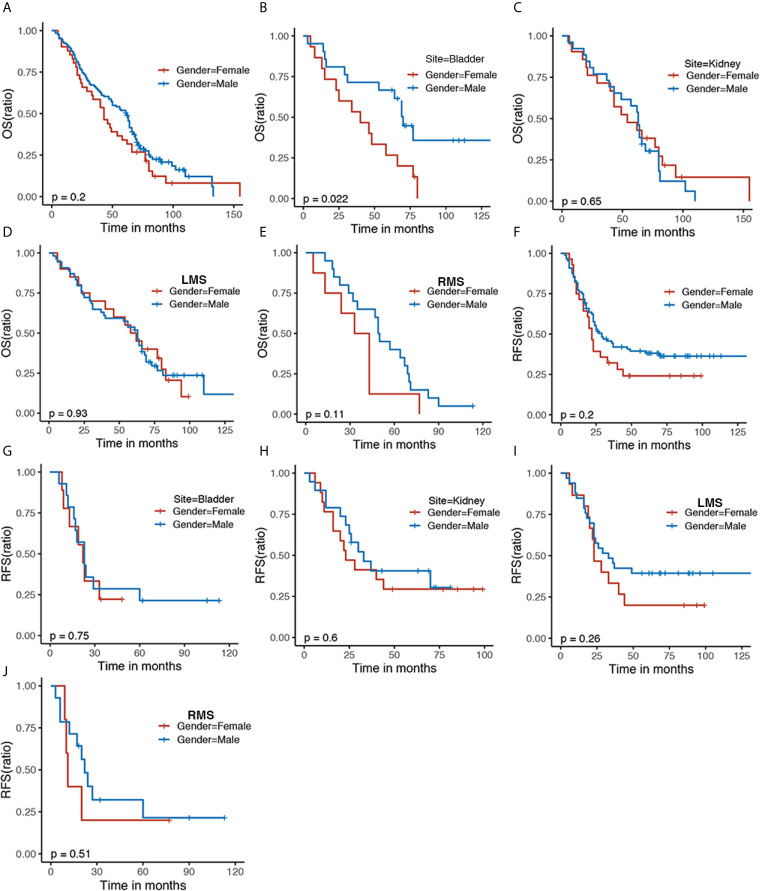
The Kaplan–Meier survival curve comparing **(A)** OS and **(F)** RFS of the female and male patients with sarcoma. Kaplan–Meier survival estimating **(B, C)** OS and **(G, H)** RFS of bladder and kidney sarcoma stratified by gender as well as histological subtypes including LMS **(D, I)** and RMS **(E, J)**. OS, overall survival; RFS, recurrence-free survival; LMS, leiomyosarcoma; RMS, rhabdomyosarcoma.

KM plots were generated to compare OS and RFS of patients stratified by gender. The OS of GS patients did not differ in terms of gender (**Figure 1A**). Male patients had better OS than female patients in bladder sarcoma (P = 0.022) (**Figure 1B**), which were not observed in kidney sarcoma and two histological subtypes (LMS and RMS) (**Figures 1C–E**). There were no significant differences in RFS of patients stratified by gender, which were similar to patients with kidney and bladder sarcoma and two histological subtypes (LMS and RMS) with regard to gender (**Figures 1F–J**).

### The Impact of Histological Subtype on OS and RFS of GS Patients

**Figure 2:**
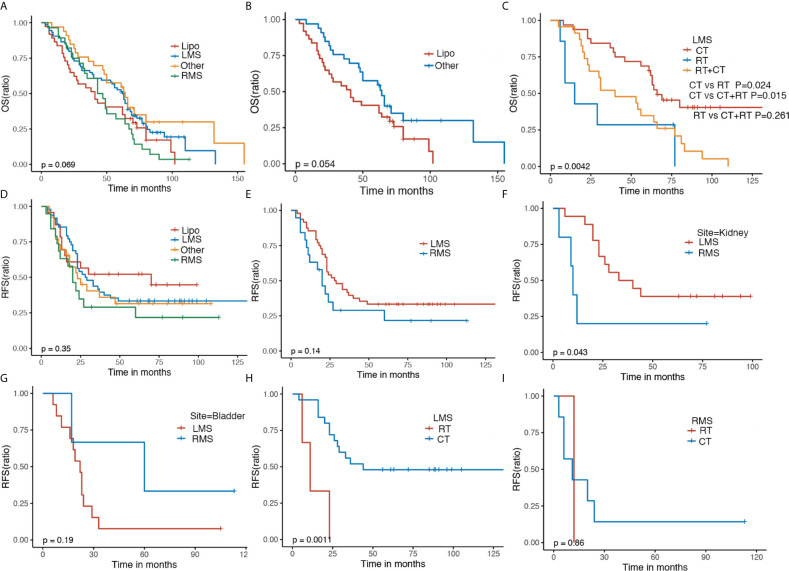
The Kaplan–Meier analyses of GS patients. The Kaplan–Meier survival estimates of **(A, B)** OS and **(D, E)** RFS stratified by histological types. The Kaplan–Meier survival estimates of **(C)** OS of LMS stratified by adjuvant treatment. The Kaplan–Meier RFS survival analysis of GS patients of kidney **(F)**, bladder **(G)**, LMS **(H)**, and RMS **(I)** stratified by adjuvant therapy. OS, overall survival; RFS, recurrence-free survival; LMS, leiomyosarcoma; RMS, rhabdomyosarcoma; Lipo, Liposarcoma; CT, chemotherapy; RT, radiotherapy.

GS patients with various histological subtypes had similar OS and RFS (**Figures 2A, D, E** **)**. Despite having no statistic difference, mild discrimination was shown between liposarcoma (Lipo) and other (P = 0.054) (**Figure 2B**). Patients with LMS undergoing chemotherapy were related to elevated OS compared with radiotherapy combined with chemotherapy or alone (**Figure 2C**). Similarly, patients with LMS were associated with favorable RFS in comparison to those with RMS in kidney sarcoma (**Figure 2F**), which did not exist in bladder sarcoma (**Figure 2G**). Furthermore, patients with LMS receiving chemotherapy were associated with optimal RFS compared with those experiencing radiotherapy (**Figure 2H**), which was not observed in those with RMS (**Figure 2I**).

### Surgery, Chemotherapy and Radiotherapy

**Figure 3:**
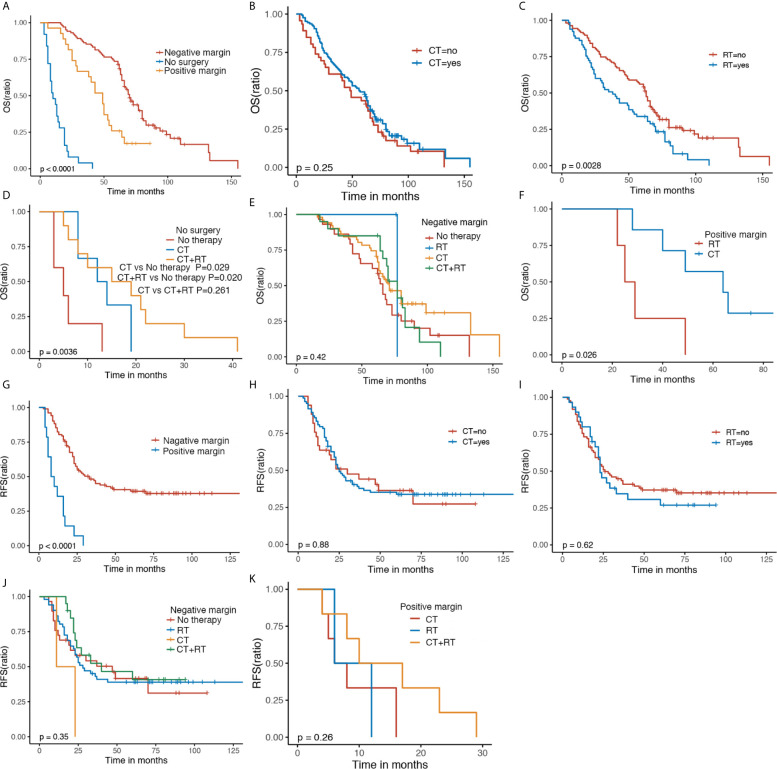
The Kaplan–Meier OS and RFS of GS patients with regard to surgery status **(A, G)**, CT **(B, H)** and RT **(C, I)**. Kaplan–Meier survival estimates of OS and RFS in GS patients with no surgery **(D)**, negative margin **(E, J)** and positive margin **(F, K)** stratified by adjuvant therapy. OS, overall survival; RFS, recurrence-free survival; CT, chemotherapy; RT, radiotherapy.

GS patients receiving surgery including negative or positive margins had a better OS in comparison to those without surgery, indicating the crucial role of surgical resection (P < 0.0001). GS patients with negative margins had a favorable OS compared with those with positive margins. (**Figure 3A**). Chemotherapy alone did not alter the OS of GS patients (**Figure 3B**). It appeared that patients who received radiotherapy had worse OS compared with those without radiotherapy (**Figure 3C**). For patients without surgery, those with chemotherapy plus radiotherapy or alone were associated with improved OS (P = 0.0036) (**Figure 3D**); for patients with negative margins, except for surgery, adjuvant therapy did not affect the OS (**Figure 3E**); and for patients with positive margins, those experiencing chemotherapy had optimal OS compared with radiotherapy (P = 0.026) (**Figure 3F**).

Patients with negative margins had preferred RFS compared with those with positive margins (P < 0.0001) (**Figure 3G**). RFS was not affected significantly by chemotherapy or radiotherapy (**Figures 3H, I** **)**. Similar RFS was observed in GS patients with negative margins following chemotherapy, radiotherapy, or combination, so was in GS patients with positive margins following chemotherapy, radiotherapy, or combination (**Figures 3J, K** **).**

### Univariate and Multivariate Analyses of OS and RFS

Results of univariate and multivariate analyses were summarized in **Tables 2**, **3**. Univariate analysis demonstrated three significant factors affecting OS of GS patients as follows: gender [HR = 0.49, 95%CI: (0.28, 0.86), P = 0.013], no surgery [HR = 37.86, 95%CI: (17.68, 81.09), P < 0.001], surgery and margin positive [HR = 4.29, 95%CI: (2.26, 8.14), P < 0.001] with surgery and margin negative as reference, and chemotherapy [HR = 0.53, 95%CI: (0.33, 0.86), P = 0.009], which remained unchanged after adjusting confounders. Meanwhile, univariate analysis demonstrated one significant factor affecting RFS: surgery and margin positive [HR = 8.54, 95%CI: (3.98, 18.34), P < 0.001] with surgery and margin negative as reference. After confounding factors were adjusted, in addition to surgery and margin positive, tumor size and symptom at presentation become significant factors. Namely, patients with tumor size ≤5 cm and those with obvious symptoms at presentation were associated with lower RFS.

**Table 2:** Univariate and multivariate analyses of overall survival in the present study.

Characteristics	Univariate analysis		Multivariate analysis (step)	
	HR [95%CI]	P-value	HR [95%CI]	P-value
**AGE**				
>50 years	Reference		Reference	
≤50 years	1.35 [0.85, 2.15]	0.207	1.36 [0.91,2.02]	0.131
**PRIMARY ORGAN**				
Bladder	Reference			
Kidney	0.84 [0.44, 1.58]	0.586	−	−
Other	1.18 [0.51, 2.73]	0.693	−	−
Paratesticular	1.01 [0.53, 1.93]	0.973	−	−
Prostate	1.22 [0.55, 2.71]	0.624	−	−
**GENDER**				
Female	Reference		Reference	
Male	0.49 [0.28, 0.86]	0.013	0.57 [0.37, 0.87]	0.009
**SYMPTOM AT PRESENTATION**				
No	Reference			
Yes	1.12 [0.43, 2.93]	0.823	−	−
**SYMPTOM DURATION**				
>1month	Reference			
≤1month	1.37 [0.86, 2.19]	0.187	−	−
**TUMOR SIZE**				
>5cm	Reference			
≤5cm	0.70 [0.40, 1.24]	0.221	−	−
**HISTOLOGICAL SUBTYPE**				
Liposarcoma	Reference		Reference	
Leiomyosarcoma	0.77 [0.45, 1.33]	0.354	0.70 [0.43, 1.15]	0.158
Other	0.70 [0.37, 1.33]	0.277	0.67 [0.37, 1.21]	0.186
Rhabdomyosarcoma	1.60 [0.82, 3.13]	0.168	1.36 [0.77, 2.40]	0.285
**METASTASIS_AT_ENTRY**				
No	Reference			
Yes	1.08 [0.69, 1.67]	0.746	−	−
**GRADE**				
High	Reference			
Low	0.81 [0.46, 1.45]	0.484	−	−
**SURGERY STATUS**				
Surgery and margin negative	Reference		Reference	
No surgery	37.86 [17.68, 81.09]	<0.001	36.88 [18.59, 73.14]	<0.001
Surgery and margin positive	4.29 [2.26, 8.14]	<0.001	3.91 [2.25, 6.81]	<0.001
**CHEMOTHERAPY**				
No	Reference		Reference	
Yes	0.53 [0.33, 0.86]	0.009	0.51 [0.34, 0.78]	0.002
**RADIOTHERAPY**				
No	Reference			
Yes	0.89 [0.56, 1.41]	0.614	−	−

Other in Primary organ included four genitourinary sites: ureter, seminal vesicles, and urethra. Other in histological subtype included Ewing’s sarcoma, primitive neuroectodermal tumor, synovial sarcoma, angiosarcoma, fibrosarcoma, malignant peripheral sheath tumor, myo-fibroblastic tumor, osteosarcoma, chondrosarcoma, and undifferentiated sarcoma.

**Table 3:** Univariate and multivariate analyses of recurrence-free survival in the present study.

Characteristics	Univariate analysis		Multivariate analysis (step)	
	HR [95%CI]	P-value	HR [95%CI]	P-value
**AGE**				
>50 years	Reference			
≤50 years	1.05 [0.62, 1.76]	0.867	−	−
**PRIMARY ORGAN**				
Bladder	Reference			
Kidney	0.73 [0.34, 1.56]	0.421	−	−
Other	0.49 [0.15, 1.59]	0.234	−	−
Paratesticular	0.62 [0.28, 1.36]	0.232	−	−
Prostate	0.95 [0.35, 2.59	0.923	−	−
**GENDER**				
Female	Reference			
Male	0.77 [0.40, 1.45]	0.414	−	−
**SYMPTOM AT PRESENTATION**				
No	Reference		Reference	
Yes	3.63 [0.85, 15.43]	0.081	3.76 [1.06, 13.32]	0.040
**SYMPTOM DURATION**				
>1month	Reference			
≤1month	1.14 [0.66, 1.96]	0.633	−	−
**TUMOR SIZE**				
>5cm	Reference		Reference	
≤5cm	1.95 [1.00, 3.79]	0.050	1.88 [1.07, 3.29]	0.028
**HISTOLOGICAL SUBTYPE**				
Liposarcoma	Reference			
Leiomyosarcoma	1.12 [0.54, 2.31]	0.766	−	−
Other	1.56 [0.69, 3.55]	0.285	−	−
Rhabdomyosarcoma	2.58 [0.98, 6.80]	0.055	−	−
**METASTASIS_AT_ENTRY**				
No	Reference			
Yes	0.97 [0.53, 1.74]	0.907	−	−
**GRADE**				
High	Reference			
Low	1.16 [0.58, 2.31]	0.669	−	−
**SURGERY STATUS**				
Surgery and margin negative	Reference		Reference	
Surgery and margin positive	8.54 [3.98, 18.34]	<0.001	6.55 [3.42, 12.54]	<0.001
**CHEMOTHERAPY**				
No	Reference			
Yes	0.90 [0.49, 1.63]	0.723	−	−
**RADIOTHERAPY**				
No	Reference			
Yes	0.63 [0.34, 1.17]	0.142	−	−

Other in Primary organ included four genitourinary sites: ureter, seminal vesicles, and urethra. Other in histological subtype included Ewing’s sarcoma, primitive neuroectodermal tumor, synovial sarcoma, angiosarcoma, fibrosarcoma, malignant peripheral sheath tumor, myo-fibroblastic tumor, osteosarcoma, chondrosarcoma, and undifferentiated sarcoma.

### Baseline Characteristics of Training and Validation Set

188 eligible patients with GS were extracted from our data according to the inclusion criteria and were randomly allocated to the training set (n = 125) and validation set (n = 63) (**Table 4**). Sociodemographic and clinicopathological features of patients in the training set and validation set were summarized, and no significant difference was observed between two sets.

**Table 4:** Clinical characteristic of patients in training set and validation set.

Characteristics	Total N = No. (%)	Training Set N = No. (%)	Validation Set N = No. (%)	P-value
**PRIMARY ORGAN**				0.351
bladder	40 (21.3)	31 (24.8)	9 (14.3)	
Kidney	49 (26.1)	28 (22.4)	21 (33.3)	
Other	13 (6.9)	9 (7.2)	4 (6.3)	
Paratesticular	56 (29.8)	36 (28.8)	20 (31.7)	
Prostate	30 (16.0)	21 (16.8)	9 (14.3)	
**GENDER**				0.295
Female	46 (24.5)	34 (27.2)	12 (19.0)	
Male	142 (75.5)	91 (72.8)	51 (81.0)	
**AGE**				0.842
>50 years	75 (39.9)	51 (40.8)	24 (38.1)	
≤50 years	113 (60.1)	74 (59.2)	39 (61.9)	
**PRESENTING SYMPTOMS**				0.283
Asymptomatic	9 (4.8)	4 (3.2)	5 (7.9)	
Symptomatic	179 (95.2)	121 (96.8)	58 (92.1)	
**SYMPTOMATIC DURATION**				0.906
>1 month	116 (61.7)	78 (62.4)	38 (60.3)	
≤1 month	72 (38.3)	47 (37.6)	25 (39.7)	
**TUMOR SIZE**				0.287
>5 cm	150 (79.8)	103 (82.4)	47 (74.6)	
≤5 cm	38 (20.2)	22 (17.6)	16 (25.4)	
**HISTOLOGICAL SUBTYPE**				0.881
Liposarcoma	38 (20.2)	27 (21.6)	11 (17.5)	
Leiomyosarcoma	77 (41.0)	51 (40.8)	26 (41.3)	
Other	37 (19.7)	23 (18.4)	14 (22.2)	
Rhabdomyosarcoma	36 (19.1)	24 (19.2)	12 (19.0)	
**METASTASIS_AT_ENTRY**				0.836
no	140 (74.5)	92 (73.6)	48 (76.2)	
yes	48 (25.5)	33 (26.4)	15 (23.8)	
**GRADE**				0.314
high	152 (80.9)	98 (78.4)	54 (85.7)	
low	36 (19.1)	27 (21.6)	9 (14.3)	
**SURGERY_STATUS**				0.585
Surgery and margin negative	106 (63.5)	69 (61.1)	37 (68.5)	
No Surgery	33 (19.8)	23 (20.4)	10 (18.5)	
Surgery and margin positive	28 (16.8)	21 (18.6)	7 (13.0)	
**CHEMOTHERAPY**				0.472
no	51 (28.3)	36 (30.5)	15 (24.2)	
yes	129 (71.7)	82 (69.5)	47 (75.8)	
**RADIOTHERAPY**				0.467
no	114 (63.3)	72 (61.0)	42 (67.7)	
yes	66 (36.7)	46 (39.0)	20 (32.3)	

Other in Primary organ included four genitourinary sites: ureter, seminal vesicles, and urethra. Other in histological subtype included Ewing′s sarcoma, primitive neuroectodermal tumor, synovial sarcoma, angiosarcoma, fibrosarcoma, malignant peripheral sheath tumor, myo-fibroblastic tumor, osteosarcoma, chondrosarcoma, and undifferentiated sarcoma.

### Prognostic Nomogram for OS

101 of 125 patients analyzed in univariate analysis were included in the multivariate analysis to characterize the risk factors of OS in the training set. As shown in **Table 5**, age, gender, surgery status, and chemotherapy were found to be associated with OS in the univariate analysis by KM method and then compared by the log-rank test (p < 0.05). Multivariate analysis demonstrated that five variates including age, gender, histological subtype, surgery status, and chemotherapy were prognostic factors for OS.

**Table 5:** Univariate and multivariate analyses of overall survival in the training cohort.

Characteristics	Univariate analysis		Multivariate analysis (step)	
	HR [95%CI]	P-value	HR [95%CI]	P-value
**AGE**				
>50 years	Reference		Reference	
≤50 years	0.98 [0.97, 0.99]	0.003	1.56 [0.91, 2.69]	0.106
**PRMARY ORGAN**				
Bladder	Reference			
Kidney	0.71 [0.36, 1.38]	0.308	−	−
Other	1.10 [0.47, 2.54]	0.831	−	−
Paratesticular	0.70 [0.35, 1.40]	0.319	−	−
Prostate	0.93 [0.41, 2.10]	0.862	−	−
**GENDER**				
Female	Reference		Reference	
Male	0.55 [0.32, 0.95]	0.031	0.51 [0.30, 0.89]	0.018
**SYMPTOM AT PRESENTATION**				
No	Reference			
Yes	0.78 [0.32, 1.90]	0.585	−	−
**SYMPTOM DURATION**				
>1month	Reference			
≤1month	1.00 [0.97, 1.05]	0.822	−	−
**TUMOR SIZE**				
>5cm	Reference			
≤5cm	1.04 [0.99, 1.09]	0.096	−	−
**HISTOLOGICAL SUBTYPE**				
Liposarcoma	Reference		Reference	
Leiomyosarcoma	0.67 [0.39, 1.15]	0.148	0.71 [0.37, 1.35]	0.294
Other	0.75 [0.40, 1.38]	0.350	0.59 [0.27, 1.29]	0.185
Rhabdomyosarcoma	1.36 [0.72, 2.57]	0.336	1.73 [0.78, 3.85]	0.179
**METASTASIS_AT_ENTRY**				
No	Reference			
Yes	1.00 [0.64, 1.57]	0.990	−	−
**GRADE**				
High	Reference			
Low	0.81 [0.46, 1.42]	0.460	−	−
**SURGERY STATUS**				
Surgery and margin negative	Reference		Reference	
No surgery	36.60 [17.27, 77.57]	<0.001	33.04 [14.37, 75.94]	<0.001
Surgery and margin positive	5.20 [2.76, 9.78]	<0.001	3.33 [1.70, 6.51]	<0.001
**CHEMOTHERAPY**				
No	Reference		Reference	
Yes	0.47 [0.30, 0.75]	0.002	0.56 [0.33, 0.95]	0.030
**RADIOTHERAPY**				
No	Reference			
Yes	0.94 [0.60, 1.48]	0.792	−	−

Other in Primary organ included four genitourinary sites: ureter, seminal vesicles, and urethra. Other in histological subtype included Ewing′s sarcoma, primitive neuroectodermal tumor, synovial sarcoma, angiosarcoma, fibrosarcoma, malignant peripheral sheath tumor, myo-fibroblastic tumor, osteosarcoma, chondrosarcoma, and undifferentiated sarcoma.

### Construction and Validation of the Nomogram

**Figure 4:**
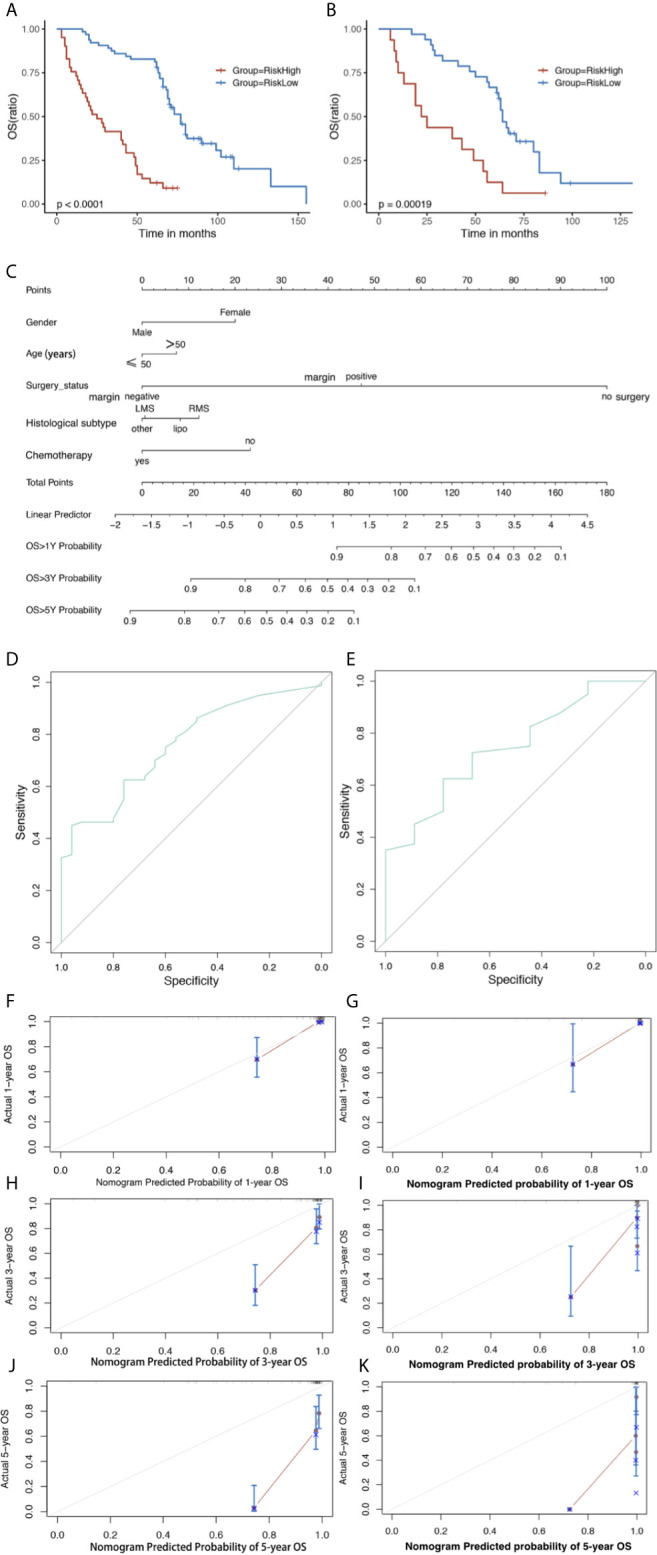
The Kaplan–Meier survival curve of risk model predicting OS in the training set **(A)** and validation set (**B**). Nomogram predicting 1-, 3- and 5-year OS **(C)** of GS patients. The nomogram is used by first giving each variable a score on its point scale. Then, add all the points and draw a vertical line from the total point scale to the axis predicting OS to obtain the probability. Receiver operating characteristic (ROC) curve of the nomogram of **(D)** Training and **(E)** validation set predicting OS. The calibration plots for the training set of **(F)** actual 1-, **(H)** 3-, and **(J)** 5-year OS; and validation set of **(G)** actual 1-, **(I)** 3-, and **(K)** 5-year OS. The 45-degree line represents an ideal match between the actual survival (Y-axis) and nomogram-predicted survival (X-axis). The perpendicular line means 95% confidence intervals. Closer distances from the points to the dashed line indicate higher prediction accuracy. OS, overall survival; AUC, area under the curve.

In the training set, all predictors of OS were integrated into the nomogram. Age, gender, histological subtype, surgery status, and chemotherapy were enrolled as predictive factors for OS (**Figure 4**). All patients in the two sets were divided into a high risk group and a low risk group according to the median risk score. OS time was obviously increased in the low risk group compared with the high risk group in the training set (log-rank P < 0.0001, **Figure 4A**). The risk stratification capability was validated by the validation set (log-rank P = 0.00019, **Figure 4B**).

Analysis of the training set showed C-index values (OS: 0.770, 95%CI: 0.760–0.772) and validation set (OS: 0.741, 95%CI: 0.740–0.765). The analysis of the training set showed AUC values (OS: 0.759, 95%CI: 0.658–0.859) and validation set (OS: 0.744, 95%CI: 0.576–0.913) (**Figures 4D, E** **)**. The calibration plots of nomogram suggested favorable accordance between predicted and observed values both in the training set and the validation set for 1-year OS (**Figures 4F, G** **)**.

## Discussion

In the present study, we found male patients with bladder sarcoma had favorable OS. Although various histological subtypes had similar prognosis, the responses to chemotherapy were different. The common subtype LMS was presumably sensitive to chemotherapy and associated with better OS and RFS following chemotherapy. LMS of kidney sarcoma was also related to favorable RFS. As an independent prognostic factor, surgical procedure obviously improved prognosis of patients. Chemotherapy served as a therapeutic choice for patients without surgery and with positive margins. Subsequently, we identified several prognostic factors and developed a nomogram to effectively predict the short-term OS, suggesting significant differentiation and calibration.

Gender was identified as an independent predictor for OS of patients with GS in the present study, which was consistent with studies enrolling patients with STS in the extremity and trunk (16, 17). Gender was related to OS, probably due to male or female predominance, different anatomical location, or biological behavior. Male predominance indeed existed in our study (72.8%). And then, we analyzed the distribution of tumor sites. The majority of female GS was located in the bladder (39.1%, 18/46) and kidney (47.8%, 22/46). By contrast, more than half of male GSs were located in paratesticular (39.4%, 56/142) and prostate (21.1%, 30/142). Bladder and kidney GSs had a poorer survival than those from other locations from a study of 3,007 patients (4). Hence, male predominance and different distribution of tumor location probably accounted for the impact of gender on OS. We also found that male patients with bladder sarcomas had optimal OS compared with female patients, which were not observed in different histological subtypes. Male patients with bladder sarcoma had a higher rate of surgery than female (88.9% *vs* 64.7%), which might be partially responsive for this difference. The precise mechanism remains unclear and further study is required to identify it.

GS is described as Lipo, followed by LMS and carcinosarcoma in a study (18). We reported that the most common histological type was LMS, followed by Lipo in the previous study (5), which was consistent with prior study on GS (1, 19). Studies on various histiotypes of GS in response to chemotherapy are scarce. LMS presents moderate sensitivity to chemotherapy, although uterine LMS has been more chemosensitive than LMS of other sites (20). In this study, patient with LMS receiving chemotherapy had better OS and RFS. Thus, it was plausible that patients with LMS could acquire survival benefit from chemotherapy. More than half of kidney sarcoma histiotypes were LMS (53.1%, 26/49), and 80.8% of those experienced chemotherapy, resulting in the lower recurrence of kidney sarcoma with LMS. This benefit was not observed in bladder sarcoma. In addition to histiotypes, chemosensitivity was probably associated with primary tumor location.

The association between tumor size and OS of patients with STS had been reported in the literatures, suggesting an inferior survival for tumor more than 5 cm or 10 cm in size (21, 22). In GS patients, we identified tumor size as an independent risk factor for recurrence, which is consistent with the results of studies (1, 23). Interestingly, we found that tumor size >5 cm was associated with a lower risk for local recurrence than those ≤5 cm, and the latter were primarily located in the bladder. We compared the recurrence of bladder GS and found that the tumors ≤5 cm in size had a higher local recurrence rate, partially due to its bladder predominance and various pathological subtypes. Distribution bias (≤5 cm, 20.2%, 38/188) partially accounted for this difference as well.

STS is currently treated in a multidisciplinary manner including surgery, chemotherapy, radiotherapy, and immunotherapy. Resection remains the cornerstone of treatment and the clinical significance of complete resection in long-term prognosis has been confirmed (24, 25). Surgery status was examined as an independent prognostic factor for OS and RFS in the present study. GS patients receiving complete resection had a better prognosis than those with incomplete resection or with no indication for operation, which was comparable to the results of a study from the Memorial Sloan-Kettering Cancer Center (1). Furthermore, surgical treatment was considered as an important determinant of survival in patients with bladder sarcoma regardless of its pathological subtypes (26). Additionally, GS patients with positive margins were related to an elevated risk for local recurrence, which was consistent with the results of a study on extremity STS (9). Based on the finding above, surgical treatment should be recommended for GS patients without contradictions and achieve negative margins to improve survival of patients. Generally, resection with 1–2 cm uninvolved tissue is considered as an ideal method, which is often limited by structural and functional constrictions (27). Meanwhile, for lack of specific clinical symptoms, sarcoma sometimes invades adjacent tissue and organ at presentation, resulting in difficulty in complete resection with macroscopic tumor clearance. Therefore, preoperatively evaluating the extent of disease and technical resectability is of importance.

In spite of the cardinal role of surgical procedure, the optimal adjuvant treatment choice for GS patients remains challenging. An early meta-analysis including 14 studies and 1,568 patients for evaluation of doxorubicin-based regimen demonstrated a significant improvement in recurrence free survival (28). However, the largest adjuvant chemotherapy trial from 1995 to 2003 showed no benefit in relapse-free survival and OS between groups (29). A study of 19 patients from Japan also uncovered no significant benefit for chemotherapy (18). Conversely, we identified chemotherapy as an independent predictor for OS in GS patients. Of all, 33 patients did not accept resection for poor general conditions or other contradictions. Chemotherapy achieved improvements in OS for these patients compared with radiation and combined therapy. Similarly, patients who had a macroscopically or microscopically positive margins could benefit from chemotherapy as well. However, chemotherapy and radiotherapy following surgery, combined or alone, did not change the prognosis of patients with negative margins. Based on the findings above, we hypothesized that chemotherapy might be a favorable option for GS patients with positive margins and without surgery and chemotherapy was not recommended for patients with negative margins. Of 188 GS patients, 106 (56.4%) patients had negative margins after surgery. Chemotherapy did not affect OS of these patients. This population bias was responsive for no remarkable effect of chemotherapy on OS of GS patients. Because prognosis of GS patients varied in different tumor sites, a conversion from single histology-specific regimens to protocols taking tumor sites into consideration probably showed promising outcomes (4). Given rarity of adult GS, there were no large clinical trials on chemotherapy available. Patients might gain benefit from the development of novel and effective agents.

In our study, local recurrence was observed in 64.7% of GS patients. Multivariate analysis identified positive margins and obvious symptom at presentation as two independent risk factors for RFS. Chemotherapy and radiotherapy did not significantly influence RFS. Furthermore, RFS for patients with negative or positive margins was not apparently altered by chemotherapy and radiotherapy. The role of chemotherapy in local recurrence was not well defined. Preoperative radiotherapy might increase the incidence of R0/R1 resection, resulting in lower RFS (30). Whether postoperative radiotherapy could influence RFS remains elusive. It appeared that patients who received radiotherapy had worse OS than those without radiotherapy. Patients with a high histological grade, positive margins and metastasis diseases were considered as candidates for radiation. This selective bias was partially responsive for poor outcomes in patients with radiotherapy.

We developed the first nomogram to predict OS and used the nomogram to predict the OS for GS patients. The performance of nomogram was evaluated by C-index and AUC of ROC, with range from 0.5 to 1.0. The C-indices for OS prediction were 0.770 in the training set and 0.741 in the validation set, respectively. Calibration plot showed a good accordance between 1-year prediction and observation.

The present study had several limitations. First, the rarity of GS might produce potential bias in terms of clinical characteristics and survival analysis, which partially explained the insufficient performance of this nomogram to predict 3- and 5-year OS. Second, retrospective nature with missing data might result in limited generalizability. Third, enrolled patients were diagnosed prior to 2010, and there were no data on neoadjuvant chemotherapy, neoadjuvant radiation, targeted therapy, and immunotherapy. A large-scale data was required to improve our nomogram for long-term prediction and external validation. Despite these limitations, our findings provided some references in the management of GS patients.

In conclusion, GS is a rare and heterogeneous tumor, and prognosis varies in different histological types following chemotherapy. LMS is sensitive to chemotherapy. Surgical resection plays a crucial role in the treatment of GS patients. Meanwhile, chemotherapy acts as a favorable option for those with positive margins and without surgical excision. A nomogram model is validated as an effective tool predicting short-term outcomes. We hope that our findings will facilitate the treatment decision-making, surveillance, and counseling.

## Data Availability Statement

The original contributions presented in the study are included in the article/supplementary material. Further inquiries can be directed to the corresponding authors.

## Ethics Statement

The studies involving human participants were reviewed and approved by the Urology Department, Urology Research Institute, West China Hospital, Sichuan University, Chengdu, Sichuan, China. The patients/participants provided their written informed consent to participate in this study.

## Author Contributions

LL and JL designed and performed the study as well as wrote the manuscript. TS, SY, JZ, and QZ performed the follow-up of patients. XF, ZJ, and YF did the data collection and analyzed the results. XW and TL designed the study and revised the manuscript. All authors reviewed the manuscript. All authors contributed to the article and approved the submitted version.

## Funding

The study was supported by the Natural Science Foundation of China (grant no. 81870513, 81470980 and 81600584) and 1.3.5 Project For Disciplines of Excellence-Clinical Research Incubation Project, West China Hospital, Sichuan University (grant no. ZY2016104, ZYJC18004, 2018HXFH049, 2021HXFH007), the Fundamental Research Funds for the Central Universities (grant no. 2017SCU11042, 2017SCU11022), A Special Supportive Program for Organ Transplantation by COTDF (grant no. 2019JYJH08), Research Funding of Sichuan Medical Association (grant no.S17056),Research Funding of Sichuan Health and Family Planning Commission (grant no. 17PJ159, 18PJ434 and 18PJ453), Chengdu Science and Technology Program (grant no. 2019-YF05-00084-SN) and Sichuan Science and Technology Program (grant no. 2019YJ0133,2019YFH0151).

## Conflict of Interest

The authors declare that the research was conducted in the absence of any commercial or financial relationships that could be construed as a potential conflict of interest.
